# Streamlining tasks and roles to expand treatment and care for HIV: randomised controlled trial protocol

**DOI:** 10.1186/1745-6215-9-21

**Published:** 2008-04-23

**Authors:** Lara R Fairall, Max O Bachmann, Merrick F Zwarenstein, Carl J Lombard, Kerry Uebel, Cloete van Vuuren, Dewald Steyn, Andrew Boulle, Eric D Bateman

**Affiliations:** 1 Knowledge Translation Unit, University of Cape Town Lung Institute, PO Box 34560, Groote Schuur 7937, South Africa; 2 Department of Medicine, University of Cape Town, PO Box 34560, Groote Schuur 7937, South Africa; 3 School of Medicine, Health Policy & Practice, University of East Anglia, Norwich NR47TJ, UK; 4 Li Ka Shing Knowledge Institute, St Michaels Hospital, Department of Health, Policy, Management and Evaluation, University of Toronto, Toronto, Canada; 5 Medical Research Council, Francie van Zijl Drive, Parowvallei, PO Box 19070, Tygerberg 7505, South Africa; 6 Department of Medicine, University of the Free State, P.O. Box 339, Bloemfontein 9300, South Africa; 7 School of Public Health and Family Medicine, University of Cape Town, Observatory 7925, South Africa

## Abstract

**Background:**

A major barrier to accessing free government-provided antiretroviral treatment (ART) in South Africa is the shortage of suitably skilled health professionals. Current South African guidelines recommend that only doctors should prescribe ART, even though most primary care is provided by nurses. We have developed an effective method of educational outreach to primary care nurses in South Africa. Evidence is needed as to whether primary care nurses, with suitable training and managerial support, can initiate and continue to prescribe and monitor ART in the majority of ART-eligible adults.

**Methods/design:**

This is a protocol for a pragmatic cluster randomised trial to evaluate the effectiveness of a complex intervention based on and supporting nurse-led antiretroviral treatment (ART) for South African patients with HIV/AIDS, compared to current practice in which doctors are responsible for initiating ART and continuing prescribing. We will randomly allocate 31 primary care clinics in the Free State province to nurse-led or doctor-led ART. Two groups of patients aged 16 years and over will be included: a) 7400 registering with the programme with CD4 counts of ≤ 350 cells/mL (mainly to evaluate treatment initiation) and b) 4900 already receiving ART (to evaluate ongoing treatment and monitoring). The primary outcomes will be time to death (in the first group) and viral suppression (in the second group). Patients' survival, viral load and health status indicators will be measured at least 6-monthly for at least one year and up to 2 years, using an existing province-wide clinical database linked to the national death register.

**Trial registration:**

Controlled Clinical Trials ISRCTN46836853

## Background

South African government health services started in 2004 to provide free ART to HIV-infected patients with CD4 counts ≤ 200 cells/μl or stage 4 AIDS, but by 2007 only a third of patients who need ART were receiving it [1]. Coverage is even lower in many other African and Asian countries [1]. The major bottleneck is due to reliance on doctors to prescribe ART, including starting treatment. Doctors are generally only available in hospitals and large urban health centres, whereas most public sector primary care clinics are staffed by nurses. Therefore better use of nurses is a compelling way to expand access and avoid delays in starting treatment. We urgently need to know whether most patients with HIV/AIDS can start and continue ART without doctors' involvement. If so, they could start treatment earlier, and thus avoid disease progression and death.

At present in South Africa only doctors may prescribe ART, in keeping with national guidelines. In the Free State province, where this trial is located, doctors initiate ART and repeat prescriptions when reviewing patients 6-monthly, with monthly visits to nurses in between. It is still widely assumed that ART is too difficult and risky to be entrusted to nurses because of drug side effects and resistance. But many eligible patients continue to die because of delays in starting ART. Our evaluation of the Free State province's ART programme to December 2005 found that, of 4570 patients followed for one year or until death, 53% died, 87% of them before they started ART [2]. However when ART was received it reduced mortality by 87%, during up to 19 months of follow up. Most patients with advanced HIV/AIDS have no contra-indications to ART, and can be managed by first line ART regimes (stavudine, lamivudine and efavirenz, or stavudine, lamivudine and nevirapine). It is thus likely that, with appropriate training and support, nurses can manage most patients effectively, leaving doctors to manage the minority at high risk or with complications.

We believe there is equipoise about whether a nurse-led system, based in primary care and with educational and managerial support, can be as effective as the current doctor-led system. On the one hand, nurses have less medical expertise than doctors and so may provide inferior care. On the other hand, if they can start treatment earlier they will probably obtain better outcomes.

A Cochrane review identified 16 randomised trials comparing primary care provided by doctors and nurses in other contexts and found that nurses could manage general medical conditions, including chronic diseases and cardiovascular risk factors, as effectively as doctors can [3]. However nurses were generally not more cost effective, because of lengthier consultations, more tests, and costs of medical supervision. None of these trials evaluated AIDS care, which is potentially more complex and risky than the types of care investigated by these trials. A Cochrane review on organisation and delivery of HIV/AIDS care highlighted the absence of trial evidence from developing countries [4]. Our own literature review found no randomised trials comparing ART care provided by doctors with ART care provided by nurses or other health workers. Recent studies from Africa [5,6] and other developed countries [7-9] described new roles for nurses in AIDS care, but they were not randomised trials and none compared doctors or nurses with other health workers.

The trial builds on two randomised trials we carried out in the same setting between 2003 and 2007. The first, Practical Approach to Lung Health in South Africa ("PALSA") trial, was a cluster randomised trial in the 40 largest primary care clinics in the Free State [10]. It evaluated a multifaceted method of educational outreach to clinic nurses based on syndromic algorithmic guidelines for integrated management of adult lung disease, building on a WHO initiative. It showed that the intervention was effective and cost effective in improving tuberculosis case detection and asthma treatment. The second, "PALSA PLUS", cluster randomised trial evaluated the extension of the guideline and training to cover HIV/AIDS care in the 15 clinics then providing ART. It demonstrated effectiveness in increasing cotrimoxazole prophylaxis and tuberculosis case detection among HIV/AIDS patients (paper submitted), which led to its adoption as a provincial programme. However at that time only doctors could initiate ART. We simultaneously conducted a cohort study of all 14267 patients enrolled on the HIV/AIDS programme to the end of 2005, discussed above [11]. These studies have demonstrated the effectiveness of our educational method and guidelines for improving quality of primary nursing care provided by nurses, the research value of these programme data, and the impact of our research on policy.

In another randomised trial in South Africa, which by April 2008 had ended recruitment but not yet reported results, patients receiving ART in two clinics were randomised to be monitored either by a HIV-trained doctor or either of two HIV-trained primary care nurses (ClinicalTrials.gov NCT00255840). Key differences between our current trial and that one are: a) our trial evaluates a complex intervention including training, staffing, and management support as well as professional substitution, and b) our trial includes all clinics and patients involved in an entire province's HIV/AIDS programme. Our search of randomised trial registers (Controlled Clinical Trials meta-register and linked registers) identified no other planned, ongoing or completed randomised trials comparing ART provision by doctors with ART provision by nurses or other health professionals.

The Free State health department has therefore decided to support this trial and to decide whether to implement nurse-led ART based on the trials' results. Despite national guidelines, its Provincial Pharmaceutical and Therapeutics Committee is legally authorised to permit nurses to prescribe Schedule 4 drugs such as antiretrovirals, and has done so for intervention clinics in this trial. The National Department of Health and the main patient advocacy group in South Africa, the Treatment Action Campaign, also support the trial and are keenly interested in the results. The timing of this trial is thus critical for policy making.

## Methods/Design

### Design

Pragmatic cluster randomised trial with clinics randomised to two parallel arms

### Aims

To compare the effectiveness of a primary care system based on nurse-led ART, with the current system based on doctor-led ART.

### Inclusion criteria

#### Clinics

All 31 nurse-staffed primary care clinics providing ART as part of the public-sector treatment program, in the Free State province, South Africa.

#### Patients

The study population will be two subgroups of HIV-infected patients aged ≤ 16 years and over enrolled with the Free State Comprehensive Care, Management and Treatment of HIV and AIDS Program. Children aged <16 are excluded because management by doctors is still considered necessary, because of complexities of drug dosages and detecting complications.

##### Subgroup 1

Patients with CD4 count ≤ 350 cells/μl and not yet receiving ART. These patients are either eligible for ART (CD4 ≤ 200) or likely to become eligible during the trial period (CD4 >200 – 350). The latter patients, with CD4s between 200 and 350 cells/μl, are included to assess the ability of nurses in intervention clinics to monitor patients up to the time they become eligible for ART and then to initiate ART promptly.

##### Subgroup 2

Patients who have already received ART for at least 6 months. This subgroup is included so as to enable evaluation of the effect of the intervention on longer term ART monitoring and re-prescriptions, while restricting the trial's follow-up period.

### Interventions

#### Control clinics

Current practice will be followed: 1. Patients eligible for ART (with CD4 ≤ 200 or stage 4 AIDS) will be referred to a doctor who will initiate and repeat prescriptions for ART and review patients every six months. Between visits to doctors, patients will be seen monthly by nurses (who may not prescribe ART) and will collect their medication. 2. Nurses will continue to use PALSA PLUS algorithmic guidelines for management of HIV/AIDS, sexually transmitted infections, and tuberculosis, having been trained to do so. These guidelines state that nurses do not prescribe ART. 3. Clinics will continue to receive routine managerial support and monitoring.

#### Intervention clinics

STRETCH is a complex intervention, to be implemented in intervention clinics, that will differ from control clinics as follows.

1. Designated nurses in each clinic will be authorised to prescribe ART. In addition to the training provided to control clinic nurses, they will receive training at their clinics covering ART prescribing, drug effects and side-effects, and use of algorithmic clinical practice guidelines including criteria for identifying patients requiring referral to a doctor. Nurses will not initiate ART in patients meeting the following criteria criteria but will refer them to the doctor (CD4 < 50, Stage 4 AIDS, previous ART, bed- or wheelchair-bound, using drugs other than cotrimoxazole or vitamins, pregnant, weight < 40 kg or body mass index >28). Doctors will also receive training about the guidelines so that they can support the nurses. These "ART nurses" will prioritise assessment and ART initiation for ART-eligible patients and carry out most treatment monitoring. Non-ART-prescribing nurses working in the ART clinics and surrounding clinics, who have received the same training as control clinic nurses, will provide routine HIV care to patients not yet eligible for ART, and refer them to ART-prescribing nurses as soon as they meet criteria for treatment. To relieve the workload of ART prescribing nurses, supportive components of ART care (apart from ART prescribing) such as drug readiness training and serial CD4 monitoring prior to ART initiation, will be decentralised to other primary care clinics that have staff trained in these components of ART care. This is intended to relieve the workload on ART prescribing nurses.

2. Managerial decisions relating to ART will be delegated to clinic managers. Clinics will receive additional managerial support through regular clinic visits by designated STRETCH co-ordinators. These arrangements, which include managerial steps to be taken, definitions of new staff roles, tips on dealing with likely problems, contact details of programme managers, and authorisation of nurse prescribing, are clearly described in the STRETCH Implementation Toolkit – a 30 page document provided to each clinic and trained nurse.

### Randomisation

Clinics were randomised to either of two arms. Randomisation was stratified by referral hospital-based "treatment site", because differences between these sites may confound clinic level ART care. However stratification was not used in one district where assessment and treatment are combined. Randomisation was carried out by the trial statistician (Lombard) before the intervention and patient recruitment started. N-Query Advisor was used to generate the allocation codes.

### Allocation concealment

Blinding and masking of patients and clinicians are not possible because, in each clinic, all eligible patients will be managed in the same way. However the trial statistician who carried out randomisation did not know the characteristics of the clinics being randomised, and the primary statistical analysis will be blinded to allocation.

### Endpoints

#### Primary outcomes

##### Subgroup 1

Time from enrolment to death. Survival analysis will be censored 12 months after the last patient has been recruited. Mortality is the most important health outcome and it is common – currently 28% of enrolled ART-eligible patients die within a year or enrolment. Deaths will be identified from programme data and by linkage with the national mortality register [2,11]. Because it is routinely recorded independently of the programme, with most deaths occurring in hospital or at home, measurement is less dependent on clinic professionals' practice, unlike adverse clinical outcomes or side effects of treatment, which require clinical skills to detect. This outcome reflects both the effectiveness of the health system in initiating treatment, and the effectiveness of ART once started.

##### Subgroup 2

Undetectable viral load (<400 copies/mL) one year after recruitment. This demonstrates continuing ART effectiveness, including adherence and treatment monitoring. Excess detectable viral loads will show whether the additional burden on nurses of initiating ART undermines the effectiveness of treatment monitoring, including dealing with poor adherence or resistance. Mortality would be a less appropriate primary outcome in this subgroup because mortality on ART is lower (17% per year [2]) and because it would probably take more than a year for suboptimal monitoring to lead to death.

#### Secondary outcomes

##### Subgroup 1

###### Process measures

time from enrolment to starting ART; proportion of patients who started ART during the study period; proportion with sputa submitted for TB screening; proportion diagnosed with tuberculosis; proportion receiving cotrimoxazole prophylaxis; nurse and doctor visits to ART programme.

###### Health measures

proportion with viral loads <400 copies/mL; baseline CD4 count of patients starting ART; changes in CD4 and weight; hospital admissions.

##### Subgroup 2

###### Process measures

proportion lost to follow-up; proportion with sputa submitted for TB screening; proportion diagnosed with tuberculosis; proportion receiving cotrimoxazole prophylaxis; nurse and doctor visits to ART programme.

###### Health measures

time from recruitment to death; changes in CD4 and weight; hospital admissions.

### Side effects: reporting and quantification

Adverse AIDS outcomes and adverse ART reactions are expected among some patients and will be monitored:

• Patients with evidence of adverse ART effects (severe rashes, lactic acidosis, severe anaemia) will be identified by the nurses and doctors monitoring them, who will record these events.

• Deaths known to ART providers will be recorded. Mortality will also be tracked by monthly linkage with deaths notified on the national population register.

• Hospital admissions, and reasons for admissions, will be continuously monitored by linkage with health department admissions data.

### Statistical analysis plan

#### Sample size

For subgroup 1, patients newly enrolled on the programme, sample size is calculated for a superiority trial (2 sided), because we hope to show decreased mortality in the nurse-led arm owing to reduced delay to starting ART. We expect to recruit at least 7400 newly enrolled patients during the first 12 months of the trial, since 4000 eligible patients were enrolled in trial clinics between 1 September and mid-December 2007. Previous programme data show that 29% of patients followed for at least a year died within one year, with an intra-clinic correlation coefficient (ICC) of 0.01. A sample size of 6000 (3000 per arm) would provide 90% power to detect a 6% difference in one-year mortality (24% vs 30%) at the 5% significance level, assuming ICC = 0.01. To cater for a 10% dropout the sample size has to be increased to 7400 in total (6000/((1-0.1)^2). Furthermore, Cox regression of time to death will have more power than comparison of proportions dying within a year.

For subgroup 2, patients already receiving ART, sample size is calculated for an equivalence trial, because we hope to show that nurse-led ART will be just as effective in suppressing HIV. We expect to recruit at least 4000 such patients since 2000 eligible patients were identified in trial clinics between 1 September and mid-December 2007. Previous programme data show that 82% of patients who had received ART for 12 months had undetectable viral loads, with an intra-clinic correlation coefficient of 0.005. A sample size of 4000 (2000 in each arm) would provide 90% power to reject the null hypothesis of non-equivalence in favor of the alternative hypothesis that the proportions patients with undetectable viral load in the two groups are equivalent using a 6% equivalence limit (i.e. within 6% of 80% in either arm), at the 5% significance level, assuming ICC = 0.005. To cater for a 10% dropout the sample size has to be increased to 4900 in total (4000/((1-0.1)^2).

#### Types of analyses

Statistical analyses will be by intention to treat. For analysis of mortality, patients with only one clinic contact will be excluded; if the proportions of patients with only one contact differ between trial arms, these analyses will be adjusted for the clinic-level proportions with only one contact. The statistical methods will be:

• Time to death: Cox proportional hazards regression

• Proportion with undetectable viral load, proportion alive after one year and other binary outcomes: Logistic regression

• CD4 and body weight: Linear regression, adjusting for baseline values

• Time to new diagnosis of tuberculosis: Cox proportional hazards regression

• Health care utilisation rates, and sputa submitted to detect tuberculosis: Poisson regression

Analyses will be at patient level and account for the stratified randomisation and intra-clinic clustering of outcomes. In secondary analyses we will also adjust for baseline confounders, if present.

#### Subgroup analyses

Patients with different CD4 levels at entry into trial (eg >200, ≤ 200 and >100, ≤ 100 cells/μl) and, among subgroup 1 patients: patients on ART at end of follow-up, patients eligible for ART but not receiving it at end of follow-up, and patients not yet eligible for ART at end of follow-up.

### Interim analyses, stopping rules and independent data monitoring committee

An independent data safety and monitoring committee, including a statistician, has been established. An interim analysis of the primary outcomes is planned one year after recruitment starts. The trial statistician (Lombard) will carry out the analysis, blinded to allocation, and report the results to the data safety and monitoring committee. If there is a highly significant (p < 0.001) difference in any primary outcome a panel comprising the researchers and Free State Department of Health managers will meet to discuss whether to terminate the trial.

### Ethical issues

The protocol was approved by the Research Ethics Committees of the University of Cape Town and University of Free State Medical Schools.

Patients with HIV/AIDS are at risk of adverse outcomes. However the treatment programme aims to improve outcomes, and there is equipoise as to whether patients in either arm would be at greater risk. According to provincial health department policy the intervention would probably be implemented incrementally in any case. The effects of the proposed research will be to randomly allocated implementation, and to provide training, managerial support and evaluation to ensure optimal implementation.

Professionals and managers in each clinic have consented to their clinic taking part in the trial. However it is not feasible to obtain patients' consent to be randomised to intervention or control arms, because randomisation will be at clinic level. Intervention- and control-type care cannot be provided within the same clinic because of the practicalities of clinic staffing, training and management, and because within-clinic randomisation would severely contaminate the trial. Even if patients preferred doctor- or nurse-led care or did not consent to take part in the trial, they would still necessarily receive the type of care that the clinic was allocated to provide [12].

Patients will not be asked for their consent for their medical records to be used for this research because it is not feasible. That is, programme managers and health professionals have insisted that obtaining such consent from all eligible patients would be a serious obstacle to widening access to HIV/AIDS care. This is because of a) the many procedures already involved in patient enrolment, b) current delays in providing effective treatment and c) the large scale of the programme, with over a thousand new enrolments per month.

However we will adhere to the ethical principles for use of medical records without patients' consent [13], as follows. The research has a clear public benefit. We have obtained approval for the study from the Research Ethics Committees of the Faculties of Health at the Universities of Cape Town and Free State, and from the lead doctors and nurses managing the programme. Use of the data for research will not influence decisions about individuals' care. The data are already being used by members of the research team for programme evaluation on behalf of the provincial health department, and for observational evaluation of ART effectiveness in the programme cohort. Only a small number of data managers have access to personal identifiers. Anonymised unlinked data (without names or national ID numbers) will be provided only to selected members of the research team – the principal investigators, the lead statistician, the data monitoring and ethics committee statistician and the health economist. There are hundreds of patients in each clinic so individuals cannot be identified from clinic names. Data with patient identifiers will be securely stored at the Lung Institute, University of Cape Town, and anonymised unlinked data will be securely stored at the University of East Anglia and the South African MRC.

## Competing interests

The authors declare that they have no competing interests.

## Authors' contributions

All authors contributed to the study design. LRF is the principal investigator. CJL is the trial statistician, and carried out the randomisation and sample size calculations. KU is the trial manager. All authors have read and approved the final manuscript.
